# Reconfigurable metasurface array for diverse retrodirective reflections and radar cross section reduction

**DOI:** 10.1515/nanoph-2024-0216

**Published:** 2024-06-17

**Authors:** Wen Yue Wei, Yan Shi, Zan Kui Meng, Ru Hui, Quan Wei Wu

**Affiliations:** School of Electronic Engineering, 47905 Xidian University, Xi’an, 710071, China

**Keywords:** retroreflection, polarization modulation, radar cross section (RCS) reduction, reconfigurable metasurface, ferrite circulators

## Abstract

Retroreflectors can scatter the arbitrarily incident wave back to incoming direction, demonstrating great potential in wireless communication. However, there are limitations in adaptive retroreflection and polarization modulation with the existing retroreflectors. In this paper, a novel metasurface array with a reconfigurable transmission line (TL) network has been proposed to flexibly achieve multiple manipulation functions of electromagnetic wave including upper half-space cross-polarized retroreflection and circularly polarized retroreflection in the diagonal planes and radar cross section (RCS) reduction. To accomplish these capabilities, a novel transmission mode for ferrite circulators has been developed, enabling precise phase control of the TL. By adjusting the operation states of the circulators, multiple phase differences between forward and reverse transmission directions including ±90° and ±180° are generated. With the obtained phase differences, the metasurface array can flexibly achieve the adaptive retroreflection fields with multiple polarization characteristics based on the spatial field superposition and the RCS reduction based on the phase cancellation. To validate the concept and feasibility of the proposed reconfigurable retrodirective metasurface, an X-band prototype has been fabricated and measured. Good agreement between the simulation and the experiment is observed to verify the effectiveness of our retrodirective design in upper half-space wave manipulation.

## 1 Introduction

During the past decades, the rapid evolution of metamaterials has been propelled by their extraordinary electromagnetic (EM) wave manipulation capabilities. Metamaterials comprising periodic or aperiodic microstructural units exhibit remarkable physical properties within specific frequency bands, breaking through the limitations of conventional materials found in nature. These composite materials can be organized into two-dimensional structures, known as metasurfaces, where unit cells with distinct electromagnetic properties are strategically placed on surfaces or interfaces. Metasurfaces offer a compelling alternative to the bulky metamaterial structures, with notable advantages including reduced physical footprint and lower losses. Consequently, metasurfaces have garnered tremendous interest from researchers and scholars globally, with a growing body of literature delving into the diverse applications. Some research efforts have explored various important applications of metasurfaces, including but not limited to polarization modulation [1], [2], [3], [4] and RCS reduction [5], [6], [7], [8], [9], [10], [11], [12], [13], [14], [15], [16], [17], [18], [19], [20], [21], [22], [23].

Retroreflectors have emerged as a powerful device for beam tracking, i.e., reflecting an incident wave from any direction back to the original direction, and thus found widespread applications in wireless communications, RFID systems, and wireless power transmission. Especially, circularly polarized (CP) retroreflectors have been widely used in satellite communication, while cross-polarized retroreflectors have demonstrated great application potential in the field of safe communication. Traditionally, retrodirective functions are achieved using corner reflector, Luneburg lens, and cat’s eyes reflector [24], [25], [26], [27], [28], [29]. However, due to their substantial size and nonplanar mechanisms, the integration with other components becomes extremely difficult, limiting their practical applications. As a planar structure, Van Atta array leverages a unique array arrangement and feeding network to enable adaptive retroreflection and has the advantages of low profile and integration friendly [30], [31], [32], [33], [34], [35], [36], [37], [38], [39], [40], [41]. The conventional Van Atta array is composed of a group of antenna elements. The retroreflection can achieve either the co- or cross-polarization wave in terms of the incoming wave, relying on the polarization characteristic of antenna element. The single-polarization antenna element generates the retroreflective wave with the same polarization as the antenna element, while the dual-polarization antenna element can achieve either co- or cross-polarized retroreflection. Nevertheless, only fixed polarization characteristic of Van Atta arrays can be achieved once the antenna elements are fabricated. Moreover, the reported cross-polarization retroreflection can be only realized for the incoming wave in two primary planes. For the incident wave in other planes, the retroreflection performance is greatly degraded owing to the superposition of two polarized components of the dual-polarized antenna element.

In recent years, there has been growing interest among researchers in achieving retroreflection using metasurfaces [42], [43]. A phase-gradient metasurface (PGM) composed of two intersecting arrays of striplines placed on both sides of a substrate has been presented to achieve the independent control over x-polarized and y-polarized retroreflections for a given incident direction [44]. However, when the incident wave direction varies, the sizes of the striplines need to be adjusted accordingly, posing significant constraints on their practical utility. In order to address this limitation, a CP retrodirective metasurface based on the spin-locked phase gradient design has been developed to enable retroreflection for various incident angles [45]. Meanwhile, an auxiliary detection-finding antenna array is introduced to determine the incident wave direction prior to retroreflection, and a motor is employed to control the rotation of each meta-atom to generate different Pancharatnam–Berry (PB) phases according to the obtained incident direction. However, the assisted operation provided by the detection-finding array significantly reduces the retroreflector’s adaptability and prevents real-time retroreflection of incoming waves.

This paper proposes a reconfigurable retrodirective metasurface array (RRMA). A unique transmission mode by using the circulator integrated into the TL has been presented to allow signals to travel between forward and reverse transmission directions of the circulators, thus resulting in desirable transmission phase modulation. By adjusting the operation states of the circulators, the cross-polarized retrodirection characteristic throughout the upper half-space can be achieved. Moreover, the CP retroreflection in two diagonal planes or RCS reduction under the irradiation of incident wave have been, respectively, demonstrated by adjusting the phase differences between the forward and reverse transmission directions of the circulators. The proof-of-concept experiments are conducted to demonstrate the effectiveness of the proposed RRMA. These diverse functionalities significantly expand the potential applications in satellite communication and safe communication of retrodirective metasurfaces.

## 2 Results

### 2.1 Basic theory of RRMA

**Figure 1:**
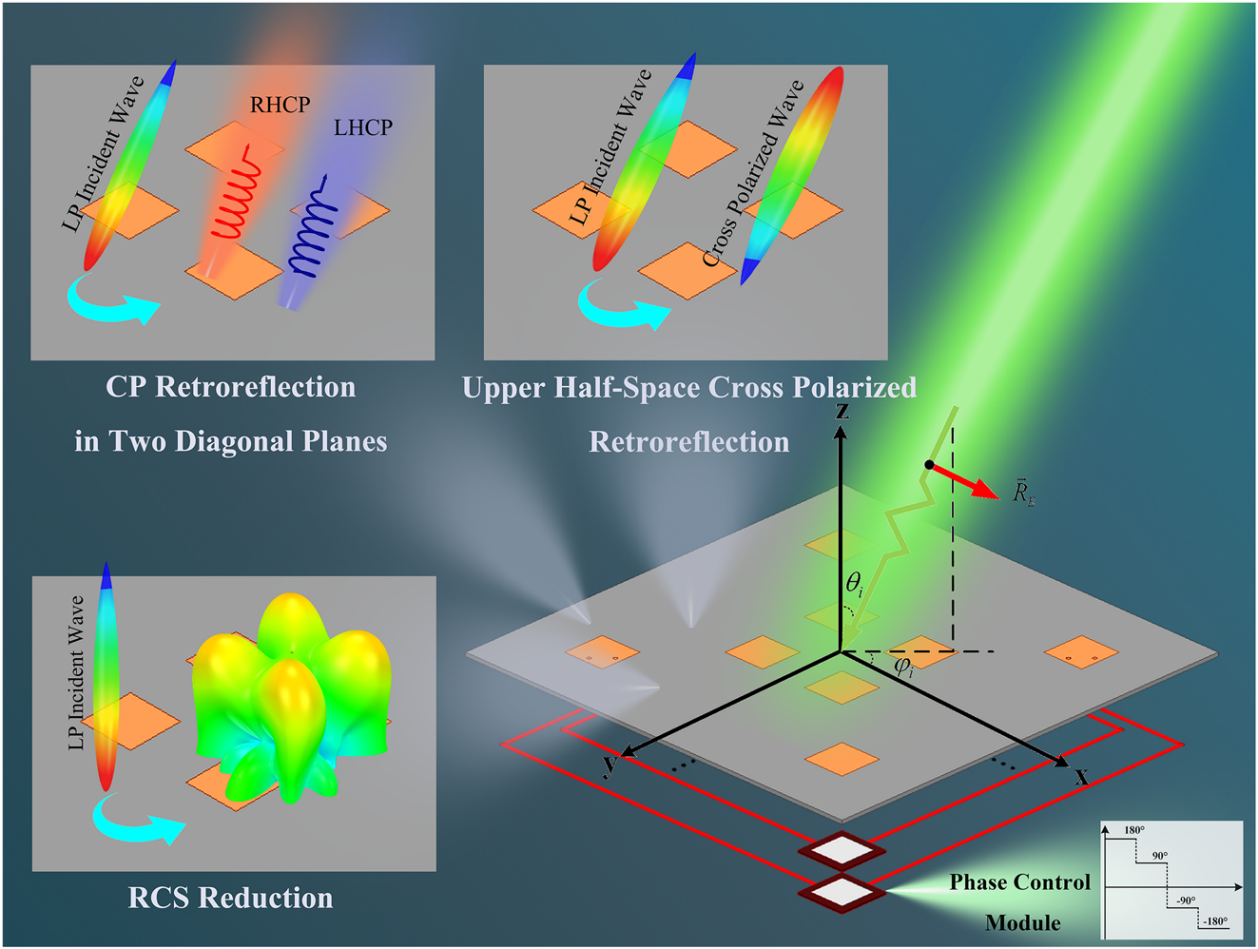
Conceptual illustration of the proposed RRMA illuminated by a linearly polarized plane wave. The phase control modules are integrated into the TLs to generate the tunable phase difference *φ*_*d*_ between the forward and reverse transmissions of each TL. The upper half-space cross-polarized retroreflection is achieved for all TLs with *φ*_*d*_ = 180°. The left-handed CP (LHCP) and right-handed CP (RHCP) retroreflections are generated in the diagonal planes for all TLs with *φ*_*d*_ = 90° and *φ*_*d*_ = −90°, respectively. The RCS reduction is realized for a half of the TLs with *φ*_*d*_ = −180° and the remaining TLs with *φ*_*d*_ = 180°.

Consider a two-dimensional metasurface array with the rotational symmetry layout about the central axis illuminated by a linearly polarized plane wave along the incident direction $\left(\theta_{i},\varphi_{i}\right)$ . Two symmetrical unit cell pairs within the array are interconnected by a TL. All TLs are of equal electrical length. Notably, each unit cell is of dual polarization, and the x-polarized port of the unit cell is connected with the y-polarized port of its symmetrical counterpart. A transmission phase control module integrated into a TL is used to manipulate the phase difference between the forward and reverse transmissions along each TL, denoted as *φ*_*d*_. As different *φ*_*d*_ is generated by the transmission phase control module, the versatile retroreflection characteristics including the cross-polarized retroreflection throughout upper half-space, the CP retroreflection in two diagonal planes, and RCS reduction are achieved, as illustrated in Figure 1.

#### 2.1.1 Upper half-space cross-polarization beam retroreflection

Assume the metasurface array in the *xoy* plane and the spacing of the unit cells in the *x* and *y* directions are *d*_*x*_ and *d*_*y*_, respectively. The coordinate origin is placed at the center of the metasurface array, which is also chosen as the phase reference point. As a plane wave with the electric field ${\overset{⇀}{E}}^{\text{Inc}}$ along the direction of $\sin\theta_{E}\cos\varphi_{E}\cdot \hat{x}+\sin\theta_{E}\sin\varphi_{E}\cdot \hat{y}+\cos\theta_{E}\cdot \hat{z}$ illuminates the metasurface array, the field received by the dual-polarized unit cell $\left(m,n\right)$ in the array can be given as

$$\begin{array}{c} {\overset{⇀}{E}}_{mn}^{\text{rec}}=I_{\text{rec}}\cdot \exp\left\{-j{\overset{⇀}{k}}_{i}\cdot {\overset{⇀}{r}}_{mn}\right\}\\ \cdot \;\left(\sin\theta_{E}\cos\varphi_{E}\cdot \hat{x}+\sin\theta_{E}\sin\varphi_{E}\cdot \hat{y}\right) \end{array}$$

where *I*_rec_ is magnitude of the received field and ${\overset{⇀}{r}}_{mn}$ is the coordinate of the center of the unit cell $\left(m,n\right)$ , ${\overset{⇀}{k}}_{i}$ is wave vector of the incident wave, and *θ*_*E*_ and *φ*_*E*_ are elevation and azimuth angles of the direction of electric field. The field received by a certain polarization port of the unit cell $\left(m,n\right)$ is transmitted through the TL and radiated by its orthogonal polarized port of the symmetric unit cell $\left(m^{\prime },n^{\prime }\right)$ . When the phase difference between the forward and reverse transmissions *φ*_*d*_ of all TLs is adjusted as π, the radiated electric field can be expressed as

$$\begin{array}{c} {\overset{⇀}{E}}_{m^{\prime }n^{\prime }}^{t}=\left[I_{\text{rec}}\cdot \exp\left\{-j{\overset{⇀}{k}}_{i}\cdot {\overset{⇀}{r}}_{mn}\right\}\right]\cdot \left[I_{\text{rec}}\cdot \exp\left\{-j{\overset{⇀}{k}}_{t}\cdot {\overset{⇀}{r}}_{m^{\prime }n^{\prime }}-j\varphi_{l}\right\}\right]\\ \cdot \;\left[\sin\theta_{E}\cos\varphi_{E}\cdot \hat{y}+\exp\left\{-j\pi \right\}\cdot \sin\theta_{E}\sin\varphi_{E}\cdot \hat{x}\right]\\ =I_{\text{rec}}^{2}\cdot \exp\left\{-j{\overset{⇀}{k}}_{i}\cdot {\overset{⇀}{r}}_{mn}-j{\overset{⇀}{k}}_{t}\cdot {\overset{⇀}{r}}_{m^{\prime }n^{\prime }}-j\varphi_{l}\right\}\\ \cdot \;\left(\sin\theta_{E}\cos\varphi_{E}\cdot \hat{y}-\sin\theta_{E}\sin\varphi_{E}\cdot \hat{x}\right) \end{array}$$

in which ${\overset{⇀}{k}}_{t}$ is wave vector of the transmitting field, *φ*_*l*_ is the phase delay of the TL, and ${\overset{⇀}{r}}_{m^{\prime }n^{\prime }}=-{\overset{⇀}{r}}_{mn}$ . Therefore, the total transmitting electric field of the metasurface array can be obtained as

(1) $$\begin{array}{c} {\overset{⇀}{E}}^{t}=I_{\text{rec}}^{2}\cdot \underset{m}{\sum }\underset{n}{\sum }\exp\left\{-j{\overset{⇀}{k}}_{i}\cdot {\overset{⇀}{r}}_{mn}+j{\overset{⇀}{k}}_{t}\cdot {\overset{⇀}{r}}_{mn}-j\varphi_{l}\right\}\\ \cdot \;\left(\sin\theta_{E}\cos\varphi_{E}\cdot \hat{y}-\sin\theta_{E}\sin\varphi_{E}\cdot \hat{x}\right) \end{array}$$

It can be seen from Equation (1) that when ${\overset{⇀}{k}}_{t}={\overset{⇀}{k}}_{i}$ , the transmitting fields of all unit cells are superposed in phase, that is, the main beam of the transmitting electric field points to the direction of the incoming wave. More importantly, the retroreflective field is orthogonal to the incident field due to ${\overset{⇀}{E}}^{t}\cdot {\overset{⇀}{E}}^{\text{Inc}}=0$ . Therefore, the cross-polarized retroreflection is achieved throughout the upper half-space under the illumination of an arbitrarily linearly polarized wave.

#### 2.1.2 Circularly polarized beam retroreflection

When the phase difference between the forward and reverse transmissions *φ*_*d*_ of all TLs is set as ±*π*/2, the transmitting field of the unit cell $\left(m^{\prime },n^{\prime }\right)$ can be written as

$$\begin{array}{c} {\overset{⇀}{E}}_{m^{\prime }n^{\prime }}^{t}=I_{\text{rec}}^{2}\cdot \exp\left(-j{\overset{⇀}{k}}_{i}\cdot {\overset{⇀}{r}}_{mn}\right)\cdot \exp\left\{-j{\overset{⇀}{k}}_{t}\cdot {\overset{⇀}{r}}_{m^{\prime }n^{\prime }}-j\varphi_{l}\right\}\\ \cdot \;\left[\sin\theta_{E}\cos\varphi_{E}\cdot \hat{y}+\exp\left\{\mp j\frac{\pi }{2}\right\}\cdot \sin\theta_{E}\sin\varphi_{E}\cdot \hat{x}\right]\\ =I_{\text{rec}}^{2}\cdot \exp\left\{-j{\overset{⇀}{k}}_{i}\cdot {\overset{⇀}{r}}_{mn}-j{\overset{⇀}{k}}_{t}\cdot {\overset{⇀}{r}}_{m^{\prime }n^{\prime }}-j\varphi_{l}\right\}\\ \cdot \;\left(\sin\theta_{E}\cos\varphi_{E}\cdot \hat{y}\mp j\sin\theta_{E}\sin\varphi_{E}\cdot \hat{x}\right) \end{array}$$

Therefore, the total retrodirective reflection field of the array can be written as

(2) $$\begin{array}{c} {\overset{⇀}{E}}^{t}=I_{\text{rec}}^{2}\cdot \underset{m}{\sum }\underset{n}{\sum }\exp\left\{-j{\overset{⇀}{k}}_{i}\cdot {\overset{⇀}{r}}_{mn}+j{\overset{⇀}{k}}_{t}\cdot {\overset{⇀}{r}}_{mn}-j\varphi_{l}\right\}\\ \cdot \;\sin\theta_{E}\left(\cos\varphi_{E}\cdot \hat{y}\mp j\sin\varphi_{E}\cdot \hat{x}\right) \end{array}$$

When $\left|\cos\varphi_{E}\right|=\left|\sin\varphi_{E}\right|$ is satisfied, i.e., *φ*_*E*_ = (2*n* + 1)*π*/4 (*n* = 0, 1, 2, 3), the total retrodirective reflection field of the metasurface array is CP. In other words, when the incident plane waves are incident in the planes with the azimuth angles of 45°, 135°, 225°, and 315°, the CP retroreflection is achieved. For *φ*_*d*_ = 90°, the retroreflective wave is LHCP, while the retroreflective wave is RHCP for *φ*_*d*_ = −90°. On the other hand, if *φ*_*E*_ = *nπ*/2 (*n* = 0, 1, 2, 3), the retroreflection field of the metasurface array is linear polarization orthogonal to the polarization of the incoming wave according to Equation (2), that is, the cross retroreflection is obtained for the incident plane wave in *xoz* and *yoz* planes. For the incident waves in other planes, the retroreflective wave becomes the elliptically polarized wave. To sum up, in the case of *φ*_*d*_ = ±90°, the retroreflections with the different polarizations in different planes are achieved for a linear polarization incident wave.

#### 2.1.3 RCS reduction

In the symmetric metasurface array, two symmetrically located unit cells $\left(m,n\right)$ and $\left(m^{\prime },n^{\prime }\right)$ are connected by two TLs. Specifically, the *x*-polarized port of the unit cell $\left(m,n\right)$ is connected to the *y*-polarized port of the unit cell $\left(m^{\prime },n^{\prime }\right)$ , while the *y*-polarized port of the unit cell $\left(m,n\right)$ is connected to the *x*-polarized of the unit cell $\left(m^{\prime },n^{\prime }\right)$ . If the *φ*_*d*_ of the two TLs are *π* and −*π*, respectively, the transmitting fields of the unit cells $\left(m^{\prime },n^{\prime }\right)$ and $\left(m,n\right)$ can be obtained as

$$\begin{array}{c} {\overset{⇀}{E}}_{m^{\prime }n^{\prime }}^{t}=I_{\text{rec}}^{2}\cdot \exp\left(-j{\overset{⇀}{k}}_{i}\cdot {\overset{⇀}{r}}_{mn}\right)\cdot \exp\left\{-j{\overset{⇀}{k}}_{t}\cdot {\overset{⇀}{r}}_{m^{\prime }n^{\prime }}-j\varphi_{l}\right\}\\ \cdot \;\left[\sin\theta_{E}\cos\varphi_{E}\cdot \hat{y}+\exp\left\{j\pi \right\}\cdot \sin\theta_{E}\sin\varphi_{E}\cdot \hat{x}\right]\\ =I_{\text{rec}}^{2}\cdot \exp\left\{-j{\overset{⇀}{k}}_{i}\cdot {\overset{⇀}{r}}_{mn}-j{\overset{⇀}{k}}_{t}\cdot {\overset{⇀}{r}}_{m^{\prime }n^{\prime }}-j\varphi_{l}\right\}\\ \cdot \;\left(\sin\theta_{E}\cos\varphi_{E}\cdot \hat{y}-\sin\theta_{E}\sin\varphi_{E}\cdot \hat{x}\right) \end{array}$$

$$\begin{array}{c} {\overset{⇀}{E}}_{mn}^{t}=I_{\text{rec}}^{2}\cdot \exp\left(-j{\overset{⇀}{k}}_{i}\cdot {\overset{⇀}{r}}_{m^{\prime }n^{\prime }}\right)\cdot \exp\left\{-j{\overset{⇀}{k}}_{t}\cdot {\overset{⇀}{r}}_{mn}-j\varphi_{l}\right\}\\ \cdot \;\left[\exp\left\{-j\pi \right\}\cdot \sin\theta_{E}\cos\varphi_{E}\cdot \hat{y}+\cdot \sin\theta_{E}\sin\varphi_{E}\cdot \hat{x}\right]\\ =I_{\text{rec}}^{2}\cdot \exp\left\{-j{\overset{⇀}{k}}_{i}\cdot {\overset{⇀}{r}}_{m^{\prime }n^{\prime }}-j{\overset{⇀}{k}}_{t}\cdot {\overset{⇀}{r}}_{mn}-j\varphi_{l}\right\}\\ \cdot \;\left(-\sin\theta_{E}\cos\varphi_{E}\cdot \hat{y}+\sin\theta_{E}\sin\varphi_{E}\cdot \hat{x}\right) \end{array}$$

The superposition of the transmitting fields of the two symmetrical unit cells along the retroreflective direction, i.e., ${\overset{⇀}{k}}_{t}={\overset{⇀}{k}}_{i}$ , is

$${\overset{⇀}{E}}_{mn}^{t}+{\overset{⇀}{E}}_{m^{\prime }n^{\prime }}^{t}=0$$

In this scenario, the fields transmitted by each pair of the symmetrical unit cells in the array are canceled out each other, achieving the RCS reduction. It is worthwhile noticing that the RCS reduction is obtained under the arbitrarily linear polarization wave illumination. Therefore, the conclusion of the RCS reduction holds for CP and elliptically polarized waves, because the CP and elliptically polarized waves can be decomposed into two orthogonal linear polarization components.

### 2.2 Methods and materials

To validate the aforementioned theoretical framework, an 8-element metasurface array system has been designed. This system primarily comprises a metasurface array with a customized transmission line network. The design steps of the metasurface array system are as follows:

(1) Designing the structure and dimensions of the unit cell to ensure good reception and transmission performance of the dual-polarized beam in the frequency band.
(2) Designing the phase control module to enable precise control of forward and reverse phase differences as described previously.
(3) Integrating phase control modules into the TL network and the resulting TL network is connected to the unit cells to achieve a complete metasurface array system.

#### 2.2.1 Unit cell design

**Figure 2:**
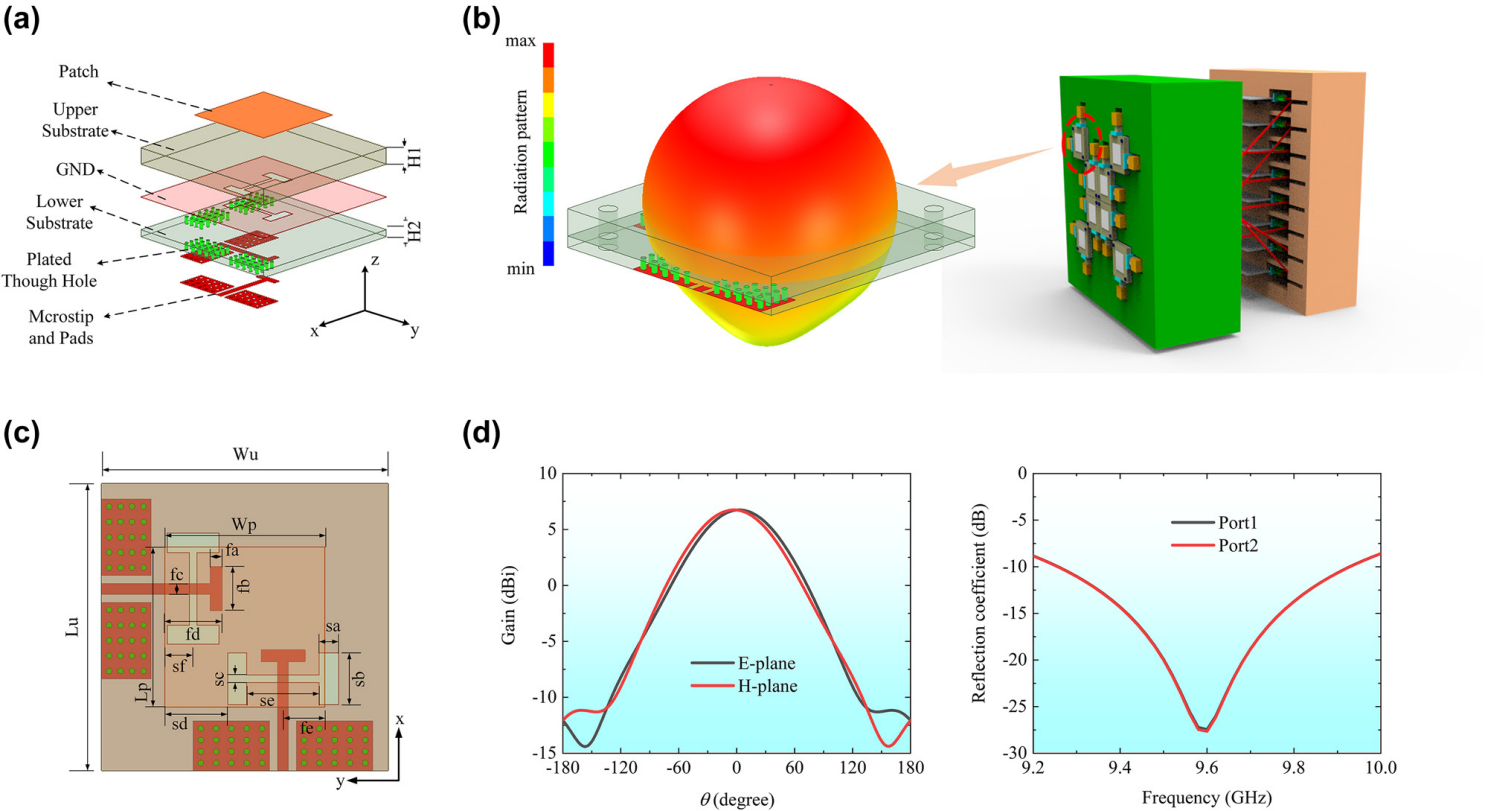
Metasurface unit cell structure and its performance including radiation patterns, gain, and reflection coefficients. (a) Schematic diagram of unit cell structure. (b) 3D radiation pattern of the unit cell in the array system. (c) Dimensioning of the unit cell. (d) Gain of the unit cell at 9.6 GHz and reflection coefficient.

As illustrated in Figure 2a, a multilayer structure is designed to receive and transmit the dual-polarized wave. A square patch is fabricated on the top surface of the upper dielectric substrate, while the microstrip line used to connect to the TL network is placed on the bottom surface of the lower dielectric substrate. The slot coupling mechanism is implemented for the reception and transmission of the wave between the top patch and the bottom microstrip line. Specifically, two I-shape slots are orthogonally etched on a ground plane, which is placed on the top surface of the lower dielectric substrate. The upper dielectric substrate is F4B material, characterized by a dielectric constant of 2.2 and a thickness of 1.5 mm, while the lower dielectric substrate employs TP-2 material with a dielectric constant of 10.2 and a thickness of 0.635 mm. The geometric parameters of the unit cell are meticulously outlined in Table 1.

**Table 1:** Detailed size of the unit cell.

Parameters	Value [mm]	Parameters	Value [mm]
Lu	15	fe	2.45
Wu	15	sa	1
Lp	8.9	sb	2.7
Wp	8.9	sc	0.4
fa	0.6	sd	3.45
fb	2.3	se	4
fc	0.57	sf	1.55
fd	3.65		

The performance of the unit cell has been analyzed by using the commercial full-wave simulation software HFSS from ANSYS Inc. The simulation results, as depicted in Figure 2d, show a gain of 6.8 dBi and almost coincident patterns in E- and H-planes. Moreover, the resonance of the unit cell occurs precisely at 9.6 GHz, and the reflection coefficients of both ports remain below −10 dB within the frequency range of 9.26–9.93 GHz, exhibiting good performance of the proposed unit cell.

#### 2.2.2 TL system and phase control module

**Figure 3:**
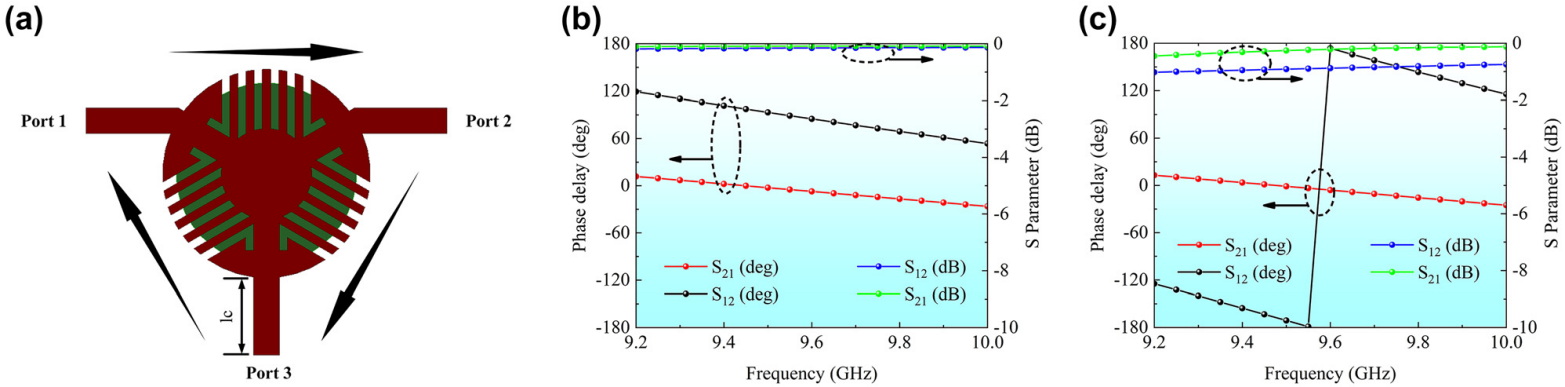
Ferrite circulator structure and its performance including transmission amplitude and transmission phase difference between the forward and reverse directions. (a) Schematic of the transmission of the ferrite circulator. (b) Transmission amplitude and phase with a phase difference of 90° between the forward and reverse directions. (c) Transmission amplitude and phase with a phase difference of 180° between the forward and reverse directions.

The key to the realization of the retroreflection metasurface array is the phase control module, with which the phase of the TL can be flexibly controlled. In the conventional microwave devices, one of the widely used phase shifters is the TL. By changing the TL length, the delay phase of the TL is adjusted. However, for a given TL, the phase difference between forward and reverse transmissions is zero. Thus, the sole TL is unsuitable for the tunable phase manipulation. As an alternative, the digital phase shifter can be used to generate the tunable phase delay. By controlling different inputs of the digital phase shifter, the desired phase delay can be achieved. However, the digital phase shifter also generates the same delay phase between forward and reverse transmissions. In this paper, a circulator has been proposed to achieve the tunable phase difference between forward and reverse transmissions for the first time. It is well known that within the circulator, electromagnetic wave propagation is tightly constrained to a specific direction. Electromagnetic waves arriving from the opposite direction find themselves effectively isolated and unable to propagate further. Different from the two-port device of the digital shifter, the three-port circulator exhibits the clockwise propagation behavior, as depicted in Figure 3a. Notably, a signal entering from port 1 can solely exit from port 2. Similarly, an input through port 2/port 3 is channeled exclusively to port 3/port 1. This property is a consequence of the distinctive magnetic attributes inherent in the ferrite material employed within the circulator. It is worthwhile pointing out that the propagation characteristic observed in the circulator arises when all three ports are appropriately matched.

In order to achieve the reverse propagation from port 2 to port 1, we design a novel transmission mode for the ferrite circulator by configuring port 3 in an open-circuit state. In this configuration, when a signal propagating from port 2 exclusively to port 3, the signal undergoes complete reflection at port 3 owing to the open-circuit state at port 3. Consequently, the reflection signal is again channeled to port 1, effectively achieving bidirectional transmission capabilities for ports 1 and 2 of the circulator. Furthermore, this configuration offers the unique advantage of generating distinct phase delays for the forward and reverse transmissions of port 1 and port 2. This property greatly simplifies the transmission process, as illustrated in Figure 3. The desired phase differences, specifically 90° and 180° in this study, can be achieved by adjusting the TL length (lc) at port 3.

The circulator’s performance has been analyzed using the HFSS. The transmission characteristics between port 1 and port 2 are given in Figure 3b and c while keeping port 3 of the circulator open and varying the lc. Remarkably, in the initial state of lc = 0.8 mm, the phase difference between port 1 and port 2 for both forward and reverse transmissions reaches precisely 90°. Meanwhile, the transmission loss is nearly negligible. When the lc is lengthened as 6.1 mm, we observe that the phase difference between port 1 and port 2 for forward and reverse transmissions expands to 180° with the transmission loss less than 1 dB.

#### 2.2.3 Metasurface array system and simulation results

**Figure 4:**
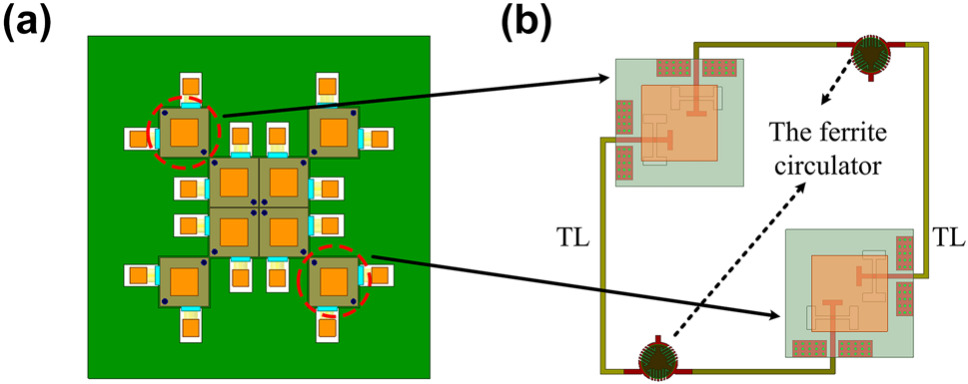
Metasurface array composed of 8 elements and a transmission line network including 8 ferrite circulators. (a) An 8-element metasurface array. (b) Schematic diagram of the connection method between the array and the TL system.

An 8-element metasurface array system, symmetrically arranged around the array center, has been constructed, as depicted in Figure 4a. Each pair of the symmetrically placed unit cells in the metasurface array is connected by two circulator integrated TLs, as shown in Figure 4b. Each of two polarization ports of a unit cell is connected to the corresponding orthogonal polarization ports of its symmetric counterpart. All TLs including the circulators form a TL network to connect all unit cells in the metasurface array. The obtained system’s performance has been investigated through extensive simulations conducted with the HFSS.

**Figure 5:**
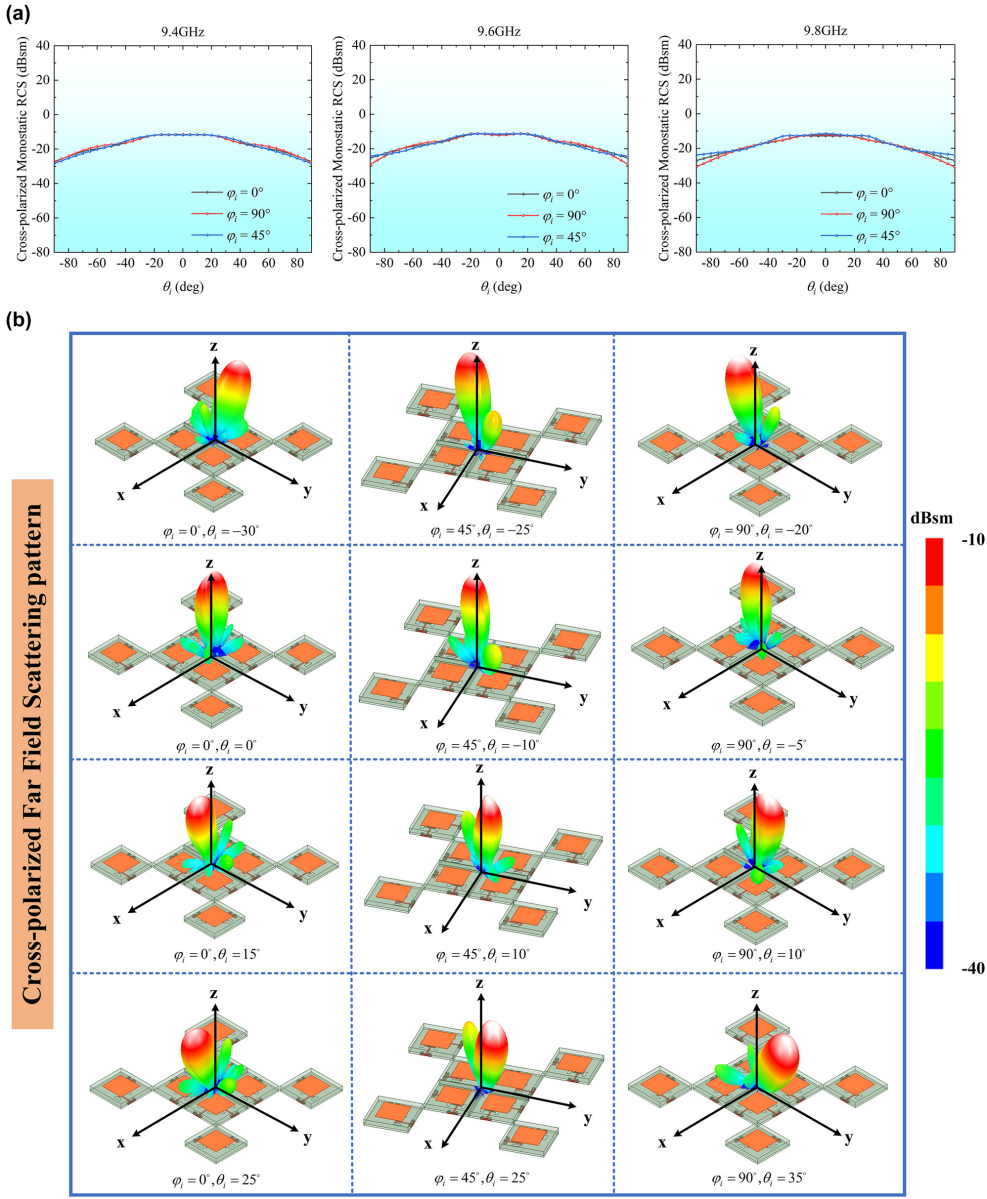
Simulation results of monostatic and 3D bistatic RCSs with the first state of the TL system. (a) Cross-polarized monostatic RCSs at 9.4 GHz, 9.6 GHz, and 9.8 GHz. (b) 3D cross-polarized bistatic RCSs in the planes of *φ*_*i*_ = 0°, *φ*_*i*_ = 45°, and *φ*_*i*_ = 90° at 9.6 GHz.

The first operation state considers the cross-polarized retroreflective performance of the proposed metasurface array within upper half-space. Here, a phase difference of 180° between forward and reverse transmissions of all TLs is generated by circulators. The simulated monostatic and bistatic RCS results for two principal planes (*φ*_*i*_ = 0° and *φ*_*i*_ = 90°) as well as the diagonal plane at *φ*_*i*_ = 45° are depicted in Figure 5. Notably, the incident wave exhibits *φ*-polarization, while the transmitted wave manifests *θ*-polarization. The transmitting beam always points toward the incident wave direction as the incoming wave direction varies. The simulations reveal exceptional performance in terms of cross-polarization retroreflection within all incident wave planes. Additionally, the array exhibits similar monostatic RCS characteristics at frequencies of 9.4 GHz, 9.6 GHz, and 9.8 GHz.

**Figure 6:**
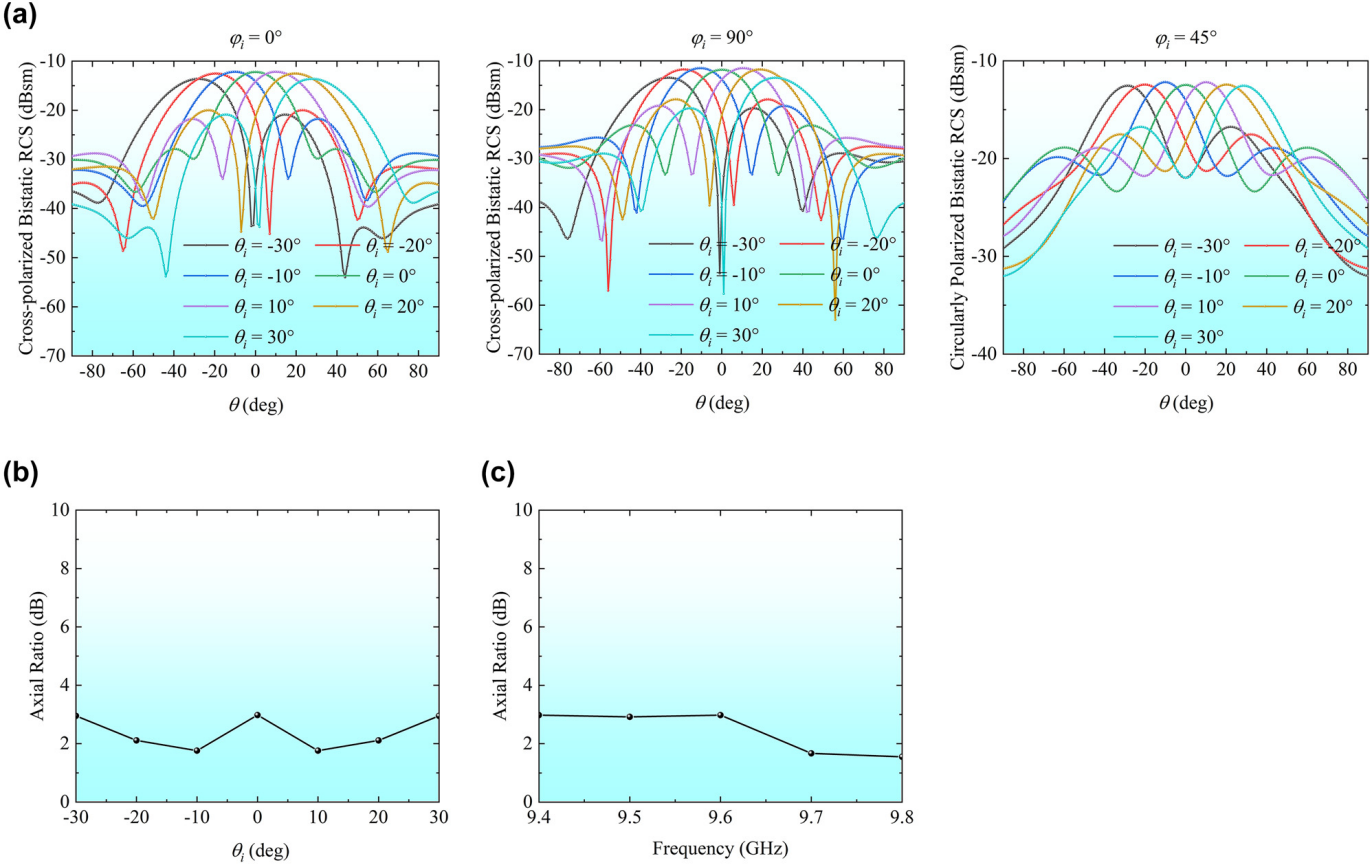
Simulation results with the second state of the TL system. (a) Bistatic RCSs in the planes of *φ*_*i*_ = 0°, *φ*_*i*_ = 90°, and *φ*_*i*_ = 45° at 9.6 GHz. (b) The axial ratios of the transmitting wave at 9.6 GHz versus incident angle in the diagonal plane. (c) The axial ratios of the transmitting wave in the direction of *θ*_*i*_ = 0° versus the operating band in the diagonal plane.

The second operation state is the retroreflections with the variable polarizations in different incident planes. In this case, a phase difference of 90° between forward and reverse transmissions of all TLs is generated by circulators. The axial ratio (AR) of the CP retroreflective field and the bistatic RCSs in the two principal planes (*φ*_*i*_ = 0° and *φ*_*i*_ = 90°) as well as the plane of *φ*_*i*_ = 45° are shown in Figure 6. Here, the AR for evaluating the CP performance is denoted as the ratio of two orthogonal components of the electric field. Notably, the primary polarization of the incident wave remains *φ*-polarization. The transmitting beam in the two main planes assumes *θ*-polarization, while the CP characteristic is exhibited in the plane of *φ*_*i*_ = 45°. Specifically, the bistatic RCS results illustrate the retrodirective reflection effect of the transmitting wave. The axial ratios of the transmitting wave at 9.6 GHz for various angles remain below the 3 dB threshold within a range of ±30°. Furthermore, we demonstrate that the axial ratios of the retrodirective reflection beams in the plane of *φ*_*i*_ = 45°, spanning frequencies from 9.4 GHz to 9.8 GHz, consistently exhibit values within the 3 dB margin.

**Figure 7:**
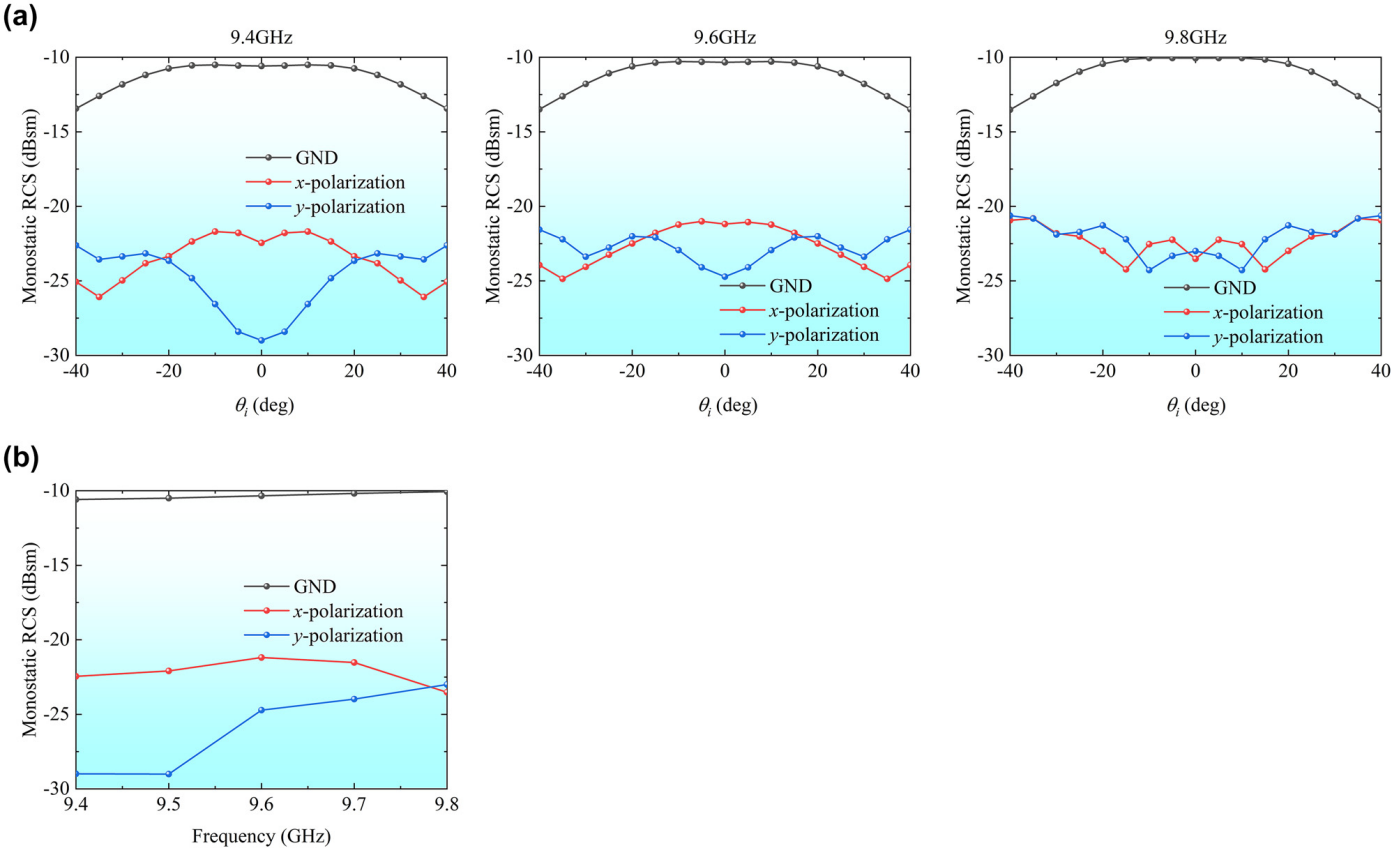
Simulation results with the third state of the TL system. (a) The monostatic RCSs at 9.4 GHz, 9.6 GHz, and 9.8 GHz for different incident directions. (b) The monostatic RCS reduction for normally incident plane wave.

The third operation state is RCS reduction, where the circulators are adjusted to achieve the phase difference of 180° between forward and reverse transmissions for a half of the TLs and the phase difference of −180° for the remaining TLs. The monostatic RCS results for the incoming waves with *x*- and *y*-polarizations are compared with those of the metallic plane of the same size as metasurface array, as shown in Figure 7. When the incident angle varies from −30° to 30°, the RCS reduction above 10 dB for two polarization waves is achieved within the band of 9.4–9.8 GHz.

### 2.3 Experimental verification

**Figure 8:**
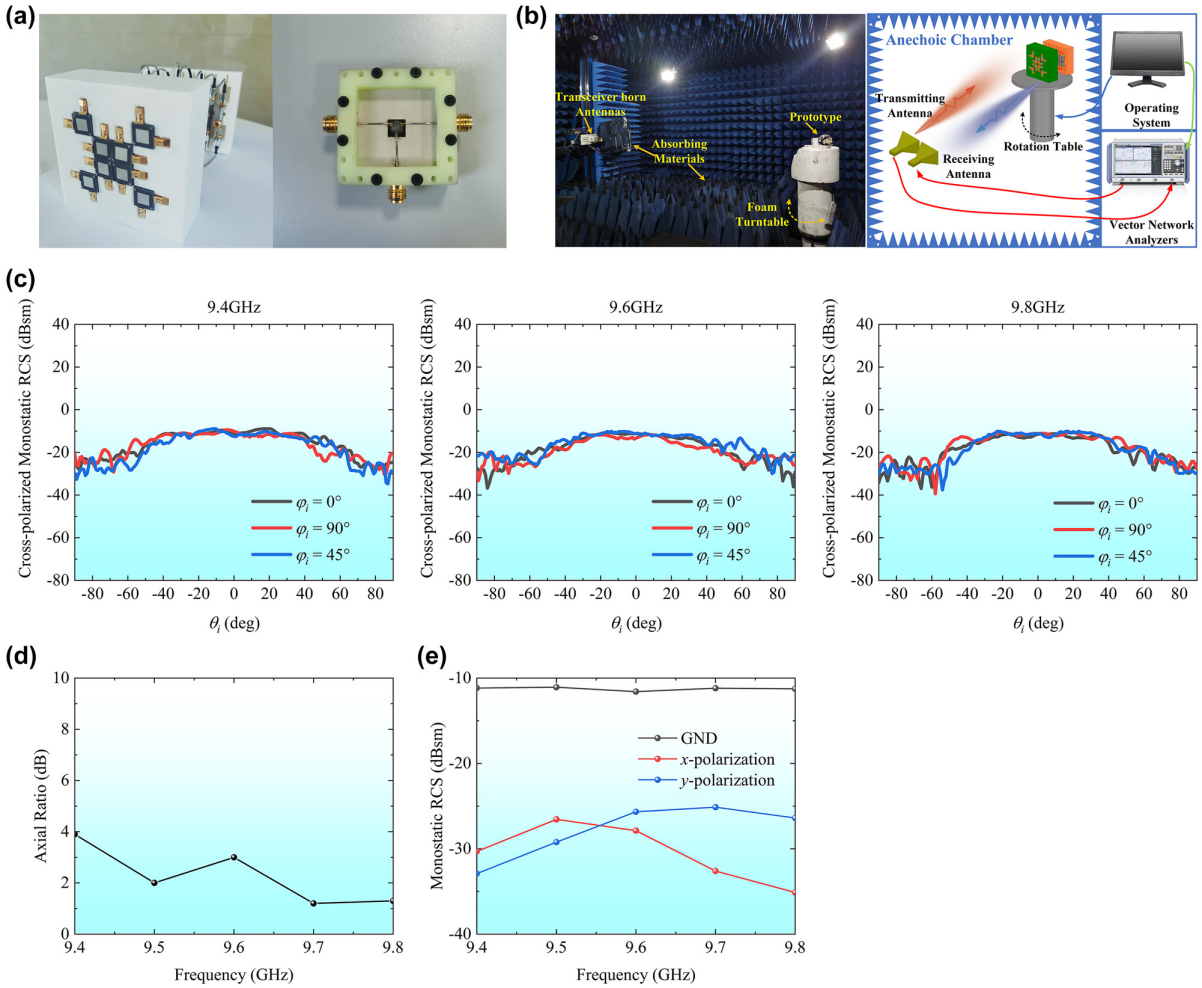
Measurement setup and measured results. (a) Fabricated reconfigurable retrodirective metasurface array and phase control module. (b) Schematic of measurement setup of the experimental environment in a microwave anechoic chamber. (c) Measured monostatic RCSs at 9.4 GHz, 9.6 GHz, and 9.8 GHz exhibiting cross-polarized retroreflection. (d) Measured axial ratios under the normal incidence. (e) Measured monostatic RCS under the normal incidence exhibiting the RCS reduction.

To validate the proposed concept, a prototype of the 8-element RRMA was fabricated, as depicted in Figure 8a. Specifically, the UIYBMC66A ferrite circulator from UIY Co., designed within the frequency range of 8–12 GHz, was employed. Three microstrip lines with the length of 17 mm were specially designed to well connect between the SMP RF connectors and the circulator, thus achieving a phase control module. It was observed that a 90° phase difference between forward and reverse transmissions was obtained when port 3 was connected to an open-circuit load. By inserting a TL section between the port 3 and the open-circuit load, the phase difference can be increased to 180°. We used 8 phase control modules and 16 RF cables of equal length to achieve the TL network, which is connected to the 8 metasurface unit cells.

The experimental setup for measuring the prototype was conducted within a microwave anechoic chamber, and its schematic diagram is illustrated in Figure 8b. In the measurement, two X-band linear-polarized horn antennas HD-100SGAH10 from Hengda Microwave were used. A horn was connected to the transmitting port of the vector network analyzer (VNA) Agilent N5244A from Agilent Technologies, while the other horn was linked to the receiving port of the VNA. To minimize unwanted coupling between the horn antennas, a wave-absorbing material was strategically placed between them. The prototype was mounted on a foam platform, which is located in the far field region of the horn antenna. The control system outside the chamber was responsible for managing the rotation of the platform, allowing for measurements of backward scattering field at various incidence angles.

The upper half-space cross-polarization retroreflection was first measured when the phase control modules were configured to the first operation state, and the receiving horn was placed in an orientation orthogonal to that of the transmitting horn. As illustrated in Figure 8c, the similar cross-polarized monostatic RCSs in three planes of *φ*_*i*_ = 0°, *φ*_*i*_ = 90°, and *φ*_*i*_ = 45° were observed at 9.4 GHz, 9.6 GHz, and 9.8 GHz, which show stable retroreflection performance within the incident angle range of ±35°. Slight fluctuations were observed in the curve. These fluctuations can be attributed to environmental factors affecting the measurements. Comparing Figures 5a and 8c, the measured RCS values align with the simulation results, verifying the cross-polarization retrodirective reflection performance.

In order to measure the CP retroreflection performance, the phase control modules were configured to the second operation state. The platform is rotated such that the azimuth angle between the prototype and the horn is 45°. The transmitting horn radiated a linearly polarized wave, while the receiving horn first received the co-polarized wave and then it was rotated 90° around its center axis to receive the cross-polarized wave. The AR was calculated by the measured co- and cross-polarized components, as shown in Figure 8d. The ARs remained within 3 dB across all frequencies except at 9.4 GHz. This observation manifests the CP retroreflection beam in the plane of *φ*_*i*_ = 45° when subjected to linearly polarized incidence. The discrepancy at 9.4 GHz can be attributed to the influence of phase errors introduced by the TL and the circulator during fabrication.

Finally, we verified the RCS reduction function of the prototype, with configuring the phase control modules to the third operation state. For comparison, the monostatic RCS of the metallic plate with the same size as the metasurface array was measured. As illustrated in Figure 8e, under the normal illuminations of *x*- and *y*-polarized waves, the backward RCS reduction of the metasurface array exceeded 10 dB across the frequency band. This observation aligns with the anticipated results from the simulation. To sum up, good agreement between the simulation and the measurement validates our proposed RRMA theory for diverse manipulation functions, extending the potential applications of the retroreflection.

## 3 Conclusions

We introduced an innovative reconfigurable retrodirective metasurface array and devised a novel transmission mode for ferrite circulators. The tunable phase difference between forward and reverse transmissions was achieved by manipulating the operation state of the circulator, which allows us to achieve upper half-space cross-polarized retroreflection, circularly polarized and cross-polarization retroreflections in different planes, and RCS reduction under the linearly polarized illumination. Our proof-of-concept experiments were given to verify the diverse retroreflection performance demonstrated by the proposed metasurface array, offering advanced functionalities of the retroreflective metasurface and enhancing the practicality and potential applications of retroreflective metasurfaces in various complicated communication and radar scenarios.
